# Long-read whole-genome sequencing of SHR rat substrains with distinct substance use phenotypes

**DOI:** 10.1007/s00335-025-10187-z

**Published:** 2025-12-22

**Authors:** Adil Abdurahaman, Paige M. Lemen, Kathleen M. Kantak, Britahny M. Baskin, Camron D. Bryant, Hao Chen

**Affiliations:** 1 Department of Pharmacology, Addition Science, and Toxicology, University of Tennessee Health Science Center, 71. S. Manassas Street, Memphis, TN 38103 USA; 2 Department of Psychological and Brain Sciences, Boston University, 64 Cummington Mall, Boston, MA 02115 USA; 3 Center for Drug Discovery, Department of Pharmaceutical Sciences, Northeastern University, 360 Huntington Avenue, Boston, MA 02115 USA

## Abstract

The Spontaneously Hypertensive Rat (SHR) is a widely used model for hypertension and, more recently, for neuropsychiatric disorders such as ADHD and Substance Use Disorder (SUD). Despite a common origin, SHR substrains from different vendors exhibit distinct behavioral phenotypes, particularly in models of SUD. This study characterizes the genomic landscape of two such substrains, SHR/NCrl and SHR/NHsd, using high-fidelity long-read sequencing to identify genetic variants associated with their phenotypic divergence. We identified over 5.8 million variants shared by both substrains compared to the reference genome. These include high-impact variants in genes involved in blood pressure regulation, such as *Ramp2* and *Npy1r*, providing a potential genetic basis for their hypertensive phenotype. We also discovered 17,618 high-confidence variants unique to each substrain. From these, we selected 500 high-quality SNP markers suitable for genetic mapping studies, such as Reduced Complexity Crosses (RCCs). Computational predictions indicated moderate-impact variants in genes involved in neuroplasticity and synaptic function. Notably, the SHR/NHsd substrain carries unique missense variants in *Ptpro* and *Tenm2*, genes linked to glutamatergic synapses and SUD-related pathways. In contrast, the SHR/NCrl substrain has unique variants in genes such as *Wwc1* and *Pcdh10*, which are involved in synaptic plasticity and neurotransmission. These findings provide a critical genomic resource for dissecting the heritable components of SUD-related behaviors.

**Supplementary Information:**

The online version contains supplementary material available at 10.1007/s00335-025-10187-z.

## Introduction

The spontaneously hypertensive rat (SHR) was established as a model for essential hypertension at Kyoto University in 1963 (Okamoto and Aoki 1963). Its development involved the selective breeding of Wistar rats with hypertension, followed by progressive sibling mating to fix the hypertensive phenotype (Okamoto and Aoki 1963). This strain was subsequently transferred to the U.S. National Institutes of Health in 1966, was fully inbred by 1969, and was then distributed worldwide. Key colonies were established by commercial vendors, including Charles River Laboratories (SHR/NCrl) in 1973 and Harlan (SHR/NHsd). Since then, the SHR has become a widely used animal model for hypertension, providing critical insights into its molecular mechanisms, as well as its associated metabolic changes and vascular remodeling (Pravenec and Kurtz 2010).

Beyond cardiovascular research, the SHR has been extensively used as a neurobiological model of Attention-Deficit/Hyperactivity Disorder (ADHD) because of its robust behavioral resemblance to the disorder, including hyperactivity, impulsivity, and attentional deficits, as well as its responsiveness to psychostimulant treatment (Sagvolden et al. 2009). These features have positioned the SHR as a valuable tool for probing the neurogenetic basis of ADHD-related traits. Nonetheless, the model does not fully capture all aspects of human ADHD, and certain behavioral inconsistencies have been noted (Leffa et al. 2019). Given the clinical comorbidity and shared neural mechanisms of ADHD and Substance Use Disorder (SUD) (Rohner et al. 2023), the SHR has also become an important model for studying genetic factors contributing to addiction vulnerability (Sharp et al. 2021; Smethells et al. 2023; Chen et al. 2012; Meyer et al. 2010).

Despite their shared ancestry, SHR/NCrl and SHR/NHsd exhibit notable differences in behavioral phenotypes relevant to SUD. The SHR/NCrl substrain displays a profile of heightened vulnerability compared to the SHR/NHsd substrain. SHR/NCrl rats demonstrate greater impulsive and compulsive-like behaviors, which are key endophenotypes associated with SUD (Kantak et al. 2021). This behavioral profile is complemented by a greater reactivity to natural rewards, such as sucrose, suggesting a generally enhanced sensitivity to reinforcing stimuli. These traits extend into drug-related contexts, where SHR/NCrl rats show a greater locomotor response to acute cocaine administration. Ultimately, these behavioral precursors manifest in more robust cocaine self-administration, with SHR/NCrl rats demonstrating greater drug taking and seeking behaviors compared to SHR/NHsd rats (Vendruscolo et al. 2009; Kantak et al. 2021). Moreover, substrain differences in vulnerability were observed across multiple psychostimulants, including cocaine, amphetamine, and methylphenidate, when patterns of use in SHR/NCrl and SHR/NHsd rats were compared with non-SHR control strains (see Kantak, 2022 Kantak 2022 for review).

These behavioral distinctions suggest that even subtle genetic divergence between substrains can produce significant neurobehavioral variability. Identifying genetic variants that differentiate these substrains will enable the development of reliable genetic markers for reduced-complexity crosses (RCC), a forward genetic design that leverages closely related substrains with limited diversity to map quantitative trait loci associated with behavioral traits (Bryant et al. 2020). This study aims to comprehensively characterize the genetic differences between the SHR/NCrl and SHR/NHsd substrains through whole genome sequencing. We utilized the High Fidelity (HiFi) sequencing data generated on PacBio long-read systems (N50 $\approx$ 15 kb) (Wenger et al. 2019) to detect single nucleotide polymorphisms (SNPs), insertions-deletions (indels), and structural variants (SVs). We hypothesize that both shared and unique genetic variations contribute to the behavioral phenotypes of these substrains. Shared variants may predispose both substrains to certain phenotypes, such as hypertension, while unique variants are likely to contribute to the differences in SUD-related behaviors.

## Results

### Mapping of PacBio sequencing data and variant calling

We obtained 7.3 million and 7.8 million reads for the SHR/NCrl and SHR/NHsd substrains, respectively, with N50 values of 14.7 kb and 14.6 kb. Mapping these reads to the reference genome yielded a mean read depth of 39.2x for SHR/NCrl and 41.9x for SHR/NHsd (Table 1). At the chromosome level, Chr 15 showed the highest average coverage (43.3x for SHR/NCrl and 46.1x for SHR/NHsd), whereas Chr X displayed the lowest (approximately 20x in both substrains). We categorized coverage into four levels: no coverage, low (<10x), medium (10-30x), and high (>30x). While most chromosomes had less than 2% of their regions with zero coverage, Chr Y was a notable exception, with 34.7% zero coverage in both substrains. Furthermore, high-coverage regions constituted only about 25% of Chr Y, which is significantly lower than the >85% observed for other chromosomes. These findings collectively demonstrate high-quality autosomal sequencing and validate a substantially shorter SHR Y chromosome compared to the BN/NHsdMcwi reference genome.

**Table 1:** Summary of sequencing coverage of SHR/NCrl and SHR/NHsd substrains

Strain	Average coverage	Zero coverage%	Low coverage%	Medium coverage%	High coverage%
SHR/NCrl	39.21	0.01	1.50	18.40	79.02
SHR/NHsd	41.92	0.01	1.42	13.40	84.11

Low coverage is less than 10x, medium coverage is between 10x and 30x, and greater than 30x is high coverage

### Variant analysis

#### Variants shared between two substrains

Following the mapping of reads to the reference genome, we filtered the variants to retain only those with a joint quality score (Qual) exceeding 30. To identify variants common to both substrains, we selected SNPs and indels with a genotype quality (GQ) score greater than 30 for each sample and identical genotypes (homozygous or heterozygous) in both substrains. For structural variants (SVs), we exclusively retained those that passed all filtering criteria (FILTER = PASS) and exhibited the same genotype in both substrains (GQ was not provided by our pipeline).

We identified 4,666,106 SNPs (96.4% homozygous), 1,016,242 indels (98.0% homozygous), and 149,432 structural variants (SVs; 71.2% homozygous) shared between the two SHR substrains relative to the reference genome. The very low proportion of heterozygous variants, representing only 0.004% of the genome, is consistent with previous reports in other inbred rat strains (de Jong et al. 2024), confirming the highly inbred nature of these substrains and underscoring the representativeness of our dataset for these lines.

**Fig. 1:**
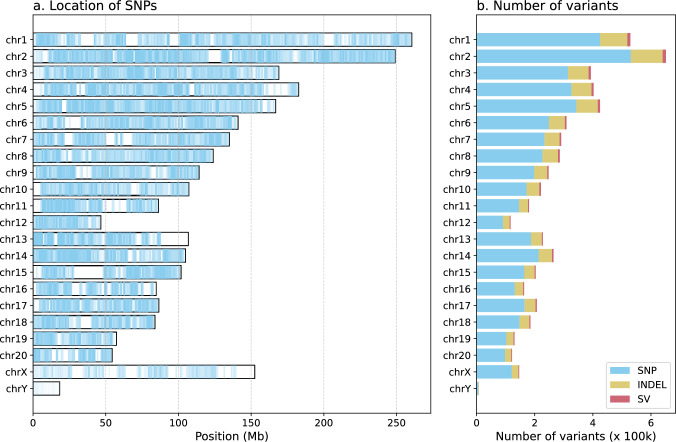
Distribution and number of shared variants. (**a**) Ideogram showing the density of shared SNPs across all chromosomes. (**b**) Bar chart illustrating the total number of shared SNPs, indels, and SVs per chromosome

A detailed chromosomal analysis of these shared variants is provided in Table 2. The shared SNPs are broadly distributed across the genome (Fig 1a). Similarly, the shared indels and SVs are also widely distributed across the genome (Fig 1b).

**Table 2:** Genomic variants found in both SHR substrains

Chr	SNP				Indel				SV	
	GQ SHR/NCrl	GQ SHR/NHsd	n	Homozygous%	GQ SHR/NCrl	GQ SHR/NHsd	n	Homozygous%	n	Homozygous%
chr1	60.5	60.5	437158	97.1	52.7	53.4	95357	98.6	14105	73.1
chr2	60.5	60.6	539533	98.4	53.1	53.9	110255	99.3	15155	73.6
chr3	60.6	60.8	322424	97.5	52.7	53.6	71844	98.8	10455	73.4
chr4	60.3	60.5	334949	97.4	52.7	53.6	70056	98.7	10100	72.3
chr5	60.8	61	346921	98.9	52.7	53.5	74195	99.5	10332	73.6
chr6	60.8	60.9	252947	98.4	53	53.8	54922	99.5	7877	73.9
chr7	60.7	60.8	236728	98.8	52.7	53.4	52143	99.3	7333	74.6
chr8	61.2	61.2	228081	99.5	52.7	53.3	53284	99.8	7985	75.5
chr9	60.8	60.9	199813	99.3	52.8	53.6	44930	99.6	6441	75
chr10	60.9	60.9	172977	99.5	51.6	52.5	44192	99.7	6890	76.6
chr11	61	61	146725	99.5	53.2	54	31282	99.7	4328	76.3
chr12	58.9	58.9	91965	99.1	50.3	51.1	22911	99.7	4406	70
chr13	60.9	60.9	188365	99.4	52.8	53.7	37497	99.8	4765	73.3
chr14	60.6	60.7	215663	99	52.8	53.7	46327	99.5	6542	76.8
chr15	60.7	60.7	165123	99.4	53	53.8	35516	99.7	5017	73.6
chr16	61	61	132217	99.5	52.8	53.7	28795	99.7	4236	77.2
chr17	60.5	60.7	167567	98.6	52.3	53.1	37894	99.4	5870	76.6
chr18	61	61	148776	99.4	52.6	53.5	33712	99.8	4871	78.2
chr19	60.6	60.7	102921	99.6	51.8	52.7	24826	99.8	4120	77.2
chr20	59.2	59.2	99104	98.9	51.7	52.4	21584	99.5	3648	70.8
chrX	54.6	56	122818	98.9	45.9	47.4	22898	99.4	3863	62.9
chrY	48.3	48.5	13331	44.9	45.1	46.6	1822	66.7	1045	12.3

*GQ* Genotype quality; Homozygous% = Number of homozygous variants/total variants $\times$ 100; *SV* Structural variant

.

#### Variants unique to each substrain

**Fig. 2:**
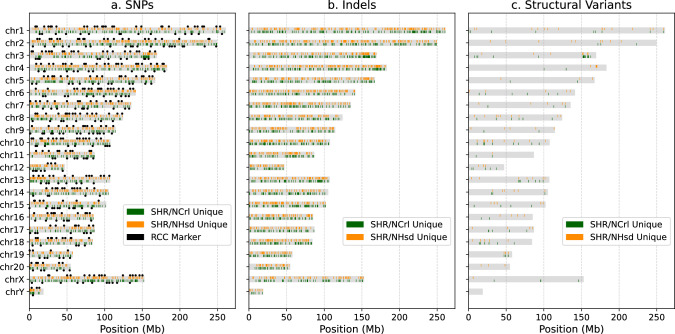
Genomic location of unique variants. Ideograms showing the distribution of unique SNPs, indels, and SVs for SHR/NCrl (green) and SHR/NHsd (orange) substrains

Despite the high degree of genetic similarity, we identified numerous unique variants in each substrain. Specifically, we found 5,059 SNPs, 2,280 indels, and 222 SVs unique to the SHR/NCrl substrain. Similarly, we identified 6,993 SNPs, 2,789 indels, and 275 SVs unique to the SHR/NHsd substrain. In total, these high-quality data revealed 17,618 genomic sites containing 215,546 bases (73.3% of the bases are due to SVs) that distinguish the two SHR substrains, representing 0.008% of the genome. Detailed counts of these strain-specific variants at the chromosomal level are provided in Table 3. The genomic locations of these variants are depicted in Fig 2.

**Table 3:** Unique variants in SHR/NCrl and SHR/NHsd substrains

	SNP				Indel				SV			
Chr	GQ (NCrl)	n (NCrl)	GQ (NHsd)	n (NHsd)	GQ (NCrl)	n (NCrl)	GQ (NHsd)	n (NHsd)	Cov (NCrl)	n (NCrl)	Cov (NHsd)	n (NHsd)
Chr1	52.2	107	53.4	136	46.8	148	47.6	169	25.8	9	24.7	19
Chr2	52.2	137	53.9	174	47.2	129	48.7	165	27.1	4	26.7	8
Chr3	55.3	2975	52.8	1610	49.9	723	48.8	362	22.4	146	24.6	62
Chr4	49.7	90	59.2	3043	47.8	118	51.2	716	24.6	4	25.6	131
Chr5	52.1	92	52.3	124	47.2	92	46.9	93	25.1	4	26.2	4
Chr6	52.3	70	53.5	106	47.5	63	48.3	103	27.8	6	25.5	7
Chr7	52.1	56	53.9	96	46.9	88	47.6	102	28.3	4	25.3	5
Chr8	51.2	69	52.8	76	46.5	68	48.4	81	21.9	8	20	8
Chr9	51.7	68	52.8	66	47.4	55	47.7	87	21.4	5	26	7
Chr10	54.5	888	54.5	849	49.3	210	50	255	25.9	29	26.8	33
Chr11	52.6	39	53.7	57	46	55	46.2	68	12	2	11.5	0
Chr12	51.6	22	52.7	40	45.4	29	45.7	35	19.7	4	20.3	6
Chr13	53.2	50	54.4	73	47.5	68	46.9	77	25.1	8	28.3	7
Chr14	52.5	48	54.1	73	49.2	63	48.5	54	22.3	5	26.3	6
Chr15	53.2	56	52.2	74	46.8	71	47	82	22.5	6	24.7	6
Chr16	51.8	31	54.2	65	46.8	54	46.8	71	23.2	5	19.5	7
Chr17	53	38	54.4	52	47	48	46.1	49	23.5	6	26.4	7
Chr18	50.4	34	52.9	62	48.5	64	49.5	62	26.5	7	27.6	5
Chr19	50.3	28	53	32	46.8	35	47.2	40	24.3	3	25.3	3
Chr20	53	24	52.7	38	46.6	32	49.2	39	17.8	0	25.5	4
ChrX	48.6	119	50.7	122	42.1	61	42.1	69	9.6	9	11	3
ChrY	49.9	18	48.5	25	48.5	6	45.5	10	14	2	19.8	2

*GQ* Genotype quality;* SV* Structural variant; *Cov* Read coverage

To identify genetic markers suitable for future genetic mapping studies in F2 crosses derived from these two substrains, we implemented a robust selection strategy. First, we excluded 7,908 strain-specific SNPs located within soft-masked repeat regions of the reference genome, which are difficult to sequence. From the remaining high-confidence SNPs, we then selected the highest-quality variant within every 3 Mb window. This approach yielded 500 high-quality markers distributed across the genome, as indicated in Fig 2a. The location and genotype of these markers are provided in Supplementary File 1.

### Functional impact of variants

We predicted the functional consequences of all genetic variants using SnpEff. We identified 1,591 high-impact variants (e.g., those that alter protein structure) present in both substrains vs the BN/NHsdMcwi reference genome. The predicted consequences of these variants are provided in Supplementary File 2. A Gene Ontology (GO) enrichment analysis of the shared high-impact variants revealed significant enrichment for terms related to ’response to stimulus’ (p_adj_ = 0.01) and ’catalytic activity’ (p_adj_ = 0.03). To further explore the function of these shared high-impact variants, we annotated them with GO terms. We found two genes involved in the regulation of blood pressure: *Ramp2*, with a variant predicted to cause a frameshift, and *Npy1r*, with a variant predicted to cause alternative splicing. We also identified several genes with high-impact variants associated with SUD-related phenotypes, such as *Grin1a* and *Shank2*. More information about these selected variants is provided in Table 4. The GO annotation of the full list of genes with high-impact variants shared by the SHR substrains and having functional relevance to blood pressure regulation and neurobehavior is provided in Supplementary File 3.

**Table 4:** Gene Ontology Annotation of Selected Genes with High Impact Variants Found in Both SHR Substrains

Symbol	Gene	Location	Variant Type	Variant Impact	Top GO Annotation
*Ace3*	Angiotensin I converting enzyme (peptidyl-dipeptidase A) 3	chr10:90948465	Indel	Splice acceptor variant	Positive regulation of systemic arterial blood pressure
*Grin2a*	Glutamate ionotropic receptor NMDA type subunit 2A	chr10:5967652	SV	Frameshift variant	Excitatory postsynaptic potential
*Npy1r*	Neuropeptide Y receptor Y1	chr16:23046785	SNP	Splice acceptor variant	Regulation of blood pressure
*Ramp2*	Receptor activity modifying protein 2	chr10:86188742	indel	Frameshift variant	Regulation of blood pressure
*Shank2*	SH3 and multiple ankyrin repeat domains 2	chr1:199561302	Indel	Splice donor variant	Structural constituent of postsynaptic density

We also identified two high-impact variants unique to SHR/NHsd. These include a SNP at chr4:170085029 that impacts the splicing of *Rerg*, a gene involved in regulating cell proliferation and hormone response, and an indel at chr19:51410953 that causes a frameshift in the *Spire2* gene, which plays a role in actin cytoskeleton organization. Additionally, the SHR/NCrl substrain harbored one unique high-impact variant in the RGD1566265 gene, which encodes the charged multivesicular body protein 1B2.

We then analyzed variants predicted to have a moderate impact (e.g., missense variants, in-frame insertions or deletions). We found that the two substrains shared 12,752 moderate-impact variants affecting 6,152 genes. No significant GO enrichment was found for these genes, most likely due to the large number of genes in this category. However, upon examining the GO annotations, we identified 29 genes with moderate-impact variants that are involved in blood pressure regulation. These include *Adamts16, Agt, Ahr, Bdkrb2, Corin, Cyp11b2, Cyp11b3, Ednra, Emilin1, Enpep, Ext2, Gpr37l1, Gucy1a1, Ier3, Kl, Lnpep, Ncf2, Nedd4l, Nos2, Npr3, Npy1r, Olr59, Ppara, Prcp, Rnpep, Scnn1g, Smtn, Umod,* and *Uts2b*. Detailed information about these variants are provided in Supplementary File 4.

We also found that SHR/NCrl had 29 unique moderate-impact variants affecting 24 genes, and SHR/NHsd had 23 unique variants affecting 18 genes. Among these 42 genes, 36 genes have GO annotations. The top three GO terms with the most literature support for these genes are provided in Table 5. Additionally, the genes *Ptpro* and *Tenm2* were both annotated with the term ’glutamatergic synapse’ in the cellular component category.

**Table 5:** Gene Ontology Annotation of Variants with Moderate Impact Unique for each SHR substrain

Substrain	Gene	Location	Variant Type	Variant Impact	Function
SHR/NCrl	*Ada*	chr3:152407994	SNP	Missense	Adenosine catabolic process; adenosine deaminase activity; inosine biosynthetic process
SHR/NCrl	*Ccn*	chr3:152498926	SNP	Missense	Integrin binding; signal transduction; cell adhesion
SHR/NCrl	*Cdh1*	chr2:71584132	SNP	Missense	Adherens junction organization; cadherin binding
SHR/NCrl	*Igsf1*	chr2:143576766	SNP	Missense	Regulation of neuron migration; cell differentiation; ossification
SHR/NCrl	*Kcns*	chr3:152840018	SNP	Missense	Delayed rectifier potassium channel activity; potassium channel regulator activity; potassium ion transport
SHR/NCrl	*Krt8*	chr7:132551011	SNP	Missense	Hair cycle; intermediate filament organization; intermediate filament-based process
SHR/NCrl	*Pabpc1*	chr3:152702509	SNP	Missense	Chromatin remodeling; cytoplasmic polyadenylation; nucleus localization
SHR/NCrl	*Pcdh1*	chr2:128620463	SNP	Mssense	Cell adhesion; calcium ion binding; homophilic cell adhesion via plasma membrane adhesion molecules
SHR/NCrl	*Semg*	chr3:152912861	SNP	Missense	Sperm capacitation; antibacterial humoral response; coagulation
SHR/NCrl	*Slc35c*	chr3:154016625, chr3:154019362	SNP	Missense	UDP-glucose transmembrane transport; antiporter activity; fucosylation
SHR/NCrl	*Tas2r11*	chr4:166253137	SNP	Missense	Bitter taste receptor activity; detection of chemical stimulus involved in sensory perception of bitter taste; G protein-coupled receptor activity
SHR/NCrl	*Thbs*	chr3:105059099	SNP	Missense	Negative regulation of angiogenesis; behavioral response to pain; blood vessel morphogenesis
SHR/NCrl	*Tmem1*	chr6:47207428	SNP	Missense; Splice region	Cell migration; DNA binding; eating behavior
SHR/NCrl	*Tmprss*	chr8:45489005	SNP	Missense	Negative regulation of growth rate; positive regulation of viral entry into host cell; protein processing
SHR/NCrl	*Trim5*	chr15:82887670	SNP	Missense	Protein ubiquitination; ubiquitin protein ligase activity; defense response to virus
SHR/NCrl	*Ttpa*	chr3:152283511	SNP	Missense	Phosphatidylinositol bisphosphate binding
SHR/NCrl	*Wfdc1*	chr3:152862447, chr3:152862454	SNP	Missense	Defense response to bacterium; serine-type endopeptidase inhibitor activity; antibacterial humoral response
SHR/NCrl	*Wfdc15*	chr3:152874500	SNP	Missense	Antibacterial humoral response; innate immune response; serine-type endopeptidase inhibitor activity
SHR/NCrl	*Wfdc*	chr3:152854715	SNP	Missense	Antibacterial humoral response; innate immune response; serine-type endopeptidase inhibitor activity
SHR/NCrl	*Wwc*	chr10:20331119, chr10:20331134, chr10:20331546	SNP	Missense	Cell migration; kinase binding; molecular adaptor activity
SHR/NCrl	*Zfp21*	chr3:158896405	SNP	Missense	RNA polymerase II cis-regulatory region sequence-specific DNA binding; regulation of DNA-templated transcription; DNA-binding transcription factor activity, RNA polymerase II-specific
SHR/NCrl	*Zfp33*	chr3:154115774	SNP	Missense	Metal ion binding
SHR/NCrl	*Zfp663*	chr3:154087069, chr3:154091712	SNP	Missense	DNA-binding transcription factor activity, RNA polymerase II-specific; RNA polymerase II transcription regulatory region sequence-specific DNA binding; metal ion binding
SHR/NHsd	*Atf7i*	chr4:169428199, chr4:169428223	SNP	Missense	Positive regulation of DNA methylation-dependent heterochromatin formation; ATP hydrolysis activity; DNA methylation
SHR/NHsd	*Atp9*	chr3:157360726	SNP	Missense	ATP binding; ATP hydrolysis activity; ATPase-coupled intramembrane lipid transporter activity
SHR/NHsd	*Dennd1*	chr3:21994795	SNP	Missense	Endocytic recycling; endocytosis; phosphatidylinositol phosphate binding
SHR/NHsd	*Dhx3*	chr1:76889283	SNP	Missense	ATP binding; ATP hydrolysis activity; RNA binding
SHR/NHsd	*Erp2*	chr4:169821565	SNP	Missense	Protein folding; response to endoplasmic reticulum stress
SHR/NHsd	*Galnt1*	chr10:41821894	SNP	Missense	Polypeptide N-acetylgalactosaminyltransferase activity; O-glycan processing; protein O-linked glycosylation
SHR/NHsd	*Gucy2*	chr4:169633362	SNP	Missense	cGMP biosynthetic process; guanylate cyclase activity; ATP binding
SHR/NHsd	*L3mbtl*	chr3:151606200	SNP	Missense	Chromatin binding; histone binding; methylated histone binding
SHR/NHsd	*Mybl*	chr3:151718406, chr3:151722579	SNP, INDEL	Missense, conservative inframe insertion	RNA polymerase II cis-regulatory region sequence-specific DNA binding; positive regulation of transcription by RNA polymerase II; DNA-binding transcription activator activity, RNA polymerase II-specific
SHR/NHsd	*Ptpr*	chr4:170277105	SNP	Missense	Protein tyrosine phosphatase activity; Wnt-protein binding; axon guidance
SHR/NHsd	*Rgs2*	chr7:67268418	SNP	Missense	G-protein alpha-subunit binding; regulation of signal transduction
SHR/NHsd	*Sgk*	chr3:151655651	SNP	Missense	Potassium channel regulator activity; protein serine/threonine kinase activity; ATP binding
SHR/NHsd	*Tenm*	chr10:20540331, chr10:20540349	SNP	Missense	Cell adhesion molecule binding; heterophilic cell-cell adhesion via plasma membrane cell adhesion molecules; protein heterodimerization activity

Aside from these highlighted high- and moderate-impact variants, most substrain-specific variants were located in intergenic or intronic regions as expected (Table 6). With the exception of one HIGH impact SV impacting an uncharacterized gene (*LOC102553880*), all strain-specific SVs are predicted to be modulators. The complete list of functional predictions for strain-specific genetic variants is provided as Supplementary File 5.

**Table 6:** Summary on the impact of substrain specific Variants

Variant impact	Count
Intergenic region	6994
Intron variant	6275
Upstream gene variant	1942
Downstream gene variant	1257
3 prime UTR variant	226
5 prime UTR variant	164
Non coding transcript exon variant	117
Synonymous variant	71
Missense variant	49
Splice region variant	26
5 prime UTR premature start codon gain variant	24
Splice donor variant	1
Splice acceptor variant	1
Frameshift variant	1
Conservative inframe insertion	1

## Discussion

This study performed PacBio whole-genome sequencing of the SHR/NCrl and SHR/NHsd substrains to elucidate the genetic basis of both their shared and divergent behavioral phenotypes. We detected more than 5.8 million variants common to both substrains, confirming their shared ancestry and providing a rich resource for investigating their hypertensive phenotype. Furthermore, we identified 17,737 high-confidence variants distinguishing SHR/NCrl from SHR/NHsd, establishing a valuable foundation for dissecting the causal genes underlying their divergent behaviors, particularly in the context of SUD. We also identified many high- and moderate-impact variants affecting genes potentially contribute to the cardiovascular phenotypes of SHR and substrain-specific behavioral phenotypes.

Compared to an earlier study that sequenced the SHR/OlaIpcv substrain at 10.7$\times$ coverage using short-read Illumina technology (Atanur et al. 2010), we detected 1.29 times as many SNPs, 2.96 times as many indels, and 10.65 times as many structural variants (SVs) shared between the two SHR substrains representing the SHR lineage. The modest increase in SNPs and indels likely reflects the greater sequencing depth of our dataset (40$\times$), which improves variant detection in low-complexity or previously undercovered regions. In contrast, the substantial increase in SV discovery primarily results from the use of long-read sequencing, which enables more accurate resolution of repetitive, rearranged, and structurally complex genomic regions often missed by short-read methods. Together, these shared variants underscore the common genetic origin of the SHR substrains and offer insights into the molecular basis of their hypertensive phenotype. Among these shared variants, we identified high-impact mutations in genes with established cardiovascular roles, including a frameshift in *Ramp2* and a splice variant in *Npy1r*. Receptor activity-modifying protein 2 (*Ramp2*) forms part of the adrenomedullin receptor complex, which mediates vasodilatory signaling and contributes to vascular homeostasis (Shindo et al. 2013; Figueira and Israel 2017). Similarly, neuropeptide Y receptor Y1 (*Npy1r*) modulates sympathetic tone and vascular resistance, and genetic variation in this pathway has been associated with blood pressure regulation in humans (Wang et al. 2009).

Further supporting a genetic basis for hypertension, we identified moderate-impact variants in a range of genes involved in blood pressure regulation through renal, vascular, and neurohormonal pathways. For instance, *Agt* encodes angiotensinogen, the precursor to angiotensin II, a central component of the renin–angiotensin system (Crowley et al. 2006). The gene *Cyp11b2*, which encodes aldosterone synthase, is responsible for the final steps of aldosterone biosynthesis, a key regulator of sodium retention and systemic pressure; genetic variants of *CYP11B2* have been associated with an elevated risk for hypertension (Sydorchuk et al. 2020). The bradykinin receptor *Bdkrb2* mediates vasodilation, and its deletion has been shown to promote salt-sensitive hypertension (Kopkan et al. 2016). Renal sodium handling is influenced by the epithelial sodium channel subunit *Scnn1g* (Büsst et al. 2011) and its regulator *Nedd4l* (Luo et al. 2009), while *Umod* also affects blood pressure through its role in sodium handling (Padmanabhan et al. 2010). Additionally, natriuretic peptide signaling is controlled by *Corin*, which activates atrial natriuretic peptide (Yan et al. 2000), and *Npr3*, which clears natriuretic peptides and thus modulates blood pressure (Saulnier et al. 2011). Vascular tone is regulated by *Ednra*, which mediates endothelin-induced vasoconstriction (Benjafield et al. 2003); *Gucy1a1*, where loss-of-function variants impair NO–cGMP signaling and increase hypertension risk (Curtis 2023); and *Nos2*, where renal inhibition of inducible nitric oxide synthase leads to elevated arterial pressure (Mattson et al. 1998). Finally, sympathetic activity is modulated by *NPY1R*, where human variants alter baroreflex and stress-induced pressure responses, influencing both systolic and diastolic blood pressure (Wang et al. 2009). Taken together, these findings indicate that the hypertensive phenotype of SHR rats provides a valuable model for elucidating multiple mechanisms underlying blood pressure regulation.

Our comprehensive comparison of the genetic variants between SHR/NHsd and SHR/NCrl revealed that substrain-specific variants were broadly distributed across the genome (Fig 2). This widespread distribution supports the idea that the limited genetic variation between the substrains is a result of the accumulation and fixation of spontaneous mutations rather than inadvertent contamination from other strains.

The primary goal of this substrain comparison was to identify genetic variants that could contribute to the behavioral differences between the SHR/NCrl and SHR/NHsd substrains, particularly their divergent responses in models of drug self-administration. This objective is supported by recent research showing that SHR/NCrl rats display greater impulsivity, compulsivity, and cocaine-seeking behaviors compared to SHR/NHsd rats, with heritability estimates for these traits ranging from 22% to 40% (Kantak et al. 2021). Although no high-impact substrain-specific variants were found in genes directly linked to SUD pathways, several moderate-impact candidates were identified that converge on the regulation of glutamatergic transmission, a core neurobiological substrate of drug-seeking behavior and reinforcement learning.

The SHR/NHsd substrain carries missense variants in *Ptpro* and *Tenm2*, both of which are annotated to the glutamatergic synapse, a critical site for neuroadaptation in SUD. Protein tyrosine phosphatase receptor type O (*Ptpro*) is a marker for midbrain dopaminergic neurons and promotes the formation of excitatory synapses, linking it to the cell type and mechanisms central to cocaine reward (Jiang et al. 2017; Xu et al. 2022). Moreover, the role of *Ptpro* in BDNF signaling and neuroinflammation connects it to established pathways of SUD-related plasticity (Gatto et al. 2013; Zhang et al. 2024). The second candidate, teneurin transmembrane protein 2 (*TENM2*), has a strong genetic association with nicotine dependence in human GWAS (Quach et al. 2020) and is functionally implicated in substance use, as its suppression in Drosophila has been shown to reduce ethanol consumption (O’Farrell et al. 2023).

In the SHR/NCrl substrain, we identified unique moderate-impact variants in *Wwc1*, *Pcdh10*, and *Ccn5*, genes central to synaptic plasticity. Among them, *Wwc1* is a particularly compelling candidate. *Wwc1* directly regulates AMPA receptor trafficking and synaptic plasticity (Stepan et al. 2022, 2024), processes critical for encoding drug-associated memories and driving compulsive drug seeking. It is enriched in the cortex and hippocampus, where *Wwc1* genotype modulates hippocampal-dependent memory (Cao et al. 2023; Muse et al. 2014). *Wwc1* dysfunction has also been linked to dopamine dysregulation (Lv et al. 2025) and to neuropsychiatric conditions involving reward circuit abnormalities, including schizophrenia (Kos et al. 2016). *Pcdh10* likewise shows strong mechanistic relevance. *Pcdh10* is highly expressed in the basolateral amygdala (Schoch et al. 2017; Hoshina et al. 2022), where it regulates excitatory synapse formation and NMDA receptor levels (Schoch et al. 2017), key determinants of plasticity underlying drug-seeking behavior. Loss of *Pcdh10* disrupts amygdalar excitatory connectivity and alters fear- and anxiety-related behaviors (Hoshina et al. 2022), traits that modulate addiction vulnerability. Moreover, *PCDH10* variants have been associated with alcohol consumption (Cheng et al. 2021), supporting its role in reward-related behaviors across substances. Finally, *Ccn5* promotes neurite outgrowth and activates Akt/ERK signaling (Ohkawa et al. 2011), pathways that govern neuronal structure and are engaged during drug-induced synaptic remodeling in reward circuits.

Collectively, these substrain-specific variants affect genes that orchestrate critical aspects of excitatory synapse structure, function, and plasticity. The convergence of substrain-specific variants on glutamatergic signaling, with *Ptpro* and *Tenm2* in SHR/NHsd and *Wwc1* and *Pcdh10* in SHR/NCrl both regulating AMPAR and NMDAR function, suggests that the two substrains may harbor genetic differences that fundamentally alter the capacity for cocaine-induced synaptic plasticity. This provides a mechanistically grounded foundation for future investigations testing whether these genetic differences causally contribute to divergent cocaine self-administration phenotypes between substrains.

Several limitations should be acknowledged. The functional consequences of the identified variants were inferred computationally and require experimental validation to establish their effects on gene function and behavior. Critically, it remains unknown whether the substrain-specific variants confer enhanced or reduced functional effects. Given that SHR/NCrl rats exhibit greater cocaine vulnerability than SHR/NHsd rats, functional studies are needed to determine whether the SHR/NCrl-specific variants (e.g., in *Wwc1* and *Pcdh10*) enhance synaptic plasticity or impair compensatory mechanisms, and conversely, whether SHR/NHsd-specific variants (e.g., in *Ptpro* and *Tenm2*) confer protective effects. For genes implicated in blood pressure regulation, variants were identified relative to the normotensive BN/NHsdMcwi reference strain; functional assays are needed to determine whether SHR alleles are causative or BN alleles are protective. Although our analysis focused on coding variants, most substrain-specific differences occurred in noncoding regions (Table 6), whose regulatory roles merit further investigation. Finally, substance use in animal models only partially captures human SUD, as key social and environmental factors are absent; thus, integration with human data remains essential for translational interpretation.

In conclusion, this genomic analysis provides a high-resolution map of genetic variation in the SHR/NCrl and SHR/NHsd substrains. We have identified an extensive set of shared variants that likely contribute to their common hypertensive phenotype, as well as a set of substrain-specific variants that may underlie their divergent behaviors related to substance use. In conjunction with the well-documented behavioral differences between these substrains, the catalog of high-confidence SNP markers established in this study offers a robust platform for applying established genetic approaches, such as RCC, to map the genetic determinants of behavioral traits, including impulsivity, compulsivity, and vulnerability to substance use.

## Methods

### DNA extraction and sequencing

Genomic DNA was extracted from striatal tissue of two adult male Spontaneously Hypertensive Rats (SHR), one purchased from Charles River (SHR/NCrl) and one purchased from Envigo/Inotiv (SHR/NHsd). Both rats were approximately 120 days old at the time of euthanasia. The University of Wisconsin DNA core facility performed the extraction and sequencing on the PacBio Sequel IIe platform, using three flow cells for each rat. The resulting raw sequencing data, provided as unmapped BAM files, were merged for each substrain before subsequent analysis.

### Genomic mapping and variant calling

For each substrain, merged reads were aligned to the mRatBN7.2 reference genome using pbmm2. Single Nucleotide Polymorphisms (SNPs) and insertions/deletions (indels) were identified with DeepVariant (v1.4.0), and joint calling was performed using GLNexus (v1.4.1). Structural variants (SVs) were called using pbsv (v2.9.0). All variants were stored in VCF format. Variants residing in genomic regions with known assembly errors as previously reported (de Jong et al. 2024) were excluded from further analysis.

### Coverage and quality analysis

Genome coverage and quality for each SHR substrain were evaluated using R (version 4.4.1). We calculated chromosome-specific and genome-wide metrics, including the total number of sequenced positions, the percentage of positions with zero, low ($\le$10x), medium (11–30x), and high (>30x) coverage. We also determined the mean, median, minimum, and maximum coverage depth, along with the standard deviation of coverage.

### Identification of high-confidence and high-impact variants

VCF data was filtered to retain only variants with a high genotype quality score (Qual $\ge$ 30). Variants were then classified as either shared between the two substrains or unique to one. Summary statistics, such as the percentage of homozygous variants and average coverage depth, were calculated.

The functional consequences of variants were predicted using SnpEff with the NCBI RefSeq gene annotation. A custom Python script parsed the annotated VCF files, selecting for variants designated with a ‘HIGH‘ or ‘MODERATE‘ impact. These variants were then categorized as shared, unique to SHR/NCrl, or unique to SHR/NHsd. The categorized variants were saved as separate CSV files for subsequent analyses.

### Functional annotation and enrichment analysis

Gene Ontology (GO) and pathway enrichment analyses were conducted on genes associated with high- and moderate-impact variants using the gprofiler2 package in R. Gene lists were tested against the *Rattus norvegicus* database. A significance threshold of p < 0.05 was applied, following multiple testing corrections using the g:SCS method. The analysis encompassed GO terms (Molecular Function, Cellular Component, Biological Process) and pathways from KEGG and Reactome.

Further functional annotation was performed using the AnnotationDbi, org.Rn.eg.db, and GO.db packages in R. Gene symbols were mapped to their corresponding GO IDs, ontology categories, and PubMed references to build a comprehensive functional profile.

### RCC marker selection

A panel of candidate markers for a RCC was selected from the set of high-confidence, unique SNPs. Selection criteria required variants to be homozygous and discordant between the substrains (genotype 1/1 in one and 0/0 in the other) with a genotype quality (GQ) of $\ge$30 in both. To prioritize markers in unique genomic regions, SNPs within soft-masked repeat regions were excluded. From this refined set, a minimal marker panel was created by selecting the single SNP with the highest average genotype quality within 3-megabase (Mb) windows. This strategy ensures an even distribution of high-quality markers across the genome for genetic mapping applications.

## Supplementary Information

Below is the link to the electronic supplementary material.

Supplementary file 1 (csv 26 KB)

Supplementary file 2 (csv 142 KB)

Supplementary file 3 (tab 7 KB)

Supplementary file 4 (tab 41 KB)

Supplementary file 5 (tab 1757 KB)

## Data Availability

The "PacBio HiFi Sequencing Data" has been deposited in the NIH SRA repository and can be accessed via the following accession ID: http://identifiers.org/bioproject/PRJNA1357335. The "Annotated Variant Calls" dataset is available in Zennodo and can be accessed using DOI: 10.5281/zenodo.17517144. The "RCC Marker Dataset" is provided as "Supplementary File 1." The "High-Impact Variant Annotation Dataset" is available as "Supplementary File 2." The "Gene Ontology Annotations for Shared High-Impact Variants" dataset is included as "Supplementary File 3." The "Gene Ontology Annotations for Shared Moderate-Impact Variant" dataset is included as "Supplementary File 4." The "Functional Predictions for Strain-Specific Genetic Variants" dataset is available as "Supplementary File 5."
